# Total Vitamin C, Ascorbic Acid, Dehydroascorbic Acid, Antioxidant Properties, and Iron Content of Underutilized and Commonly Consumed Fruits in Sri Lanka

**DOI:** 10.1155/2020/4783029

**Published:** 2020-08-20

**Authors:** Hashini I. Abeysuriya, Vajira P. Bulugahapitiya, Jayatissa Loku Pulukkuttige

**Affiliations:** ^1^Department of Chemistry, Faculty of Science, University of Ruhuna, Matara 81000, Sri Lanka; ^2^Department of Botany, Faculty of Science, University of Ruhuna, Matara 81000, Sri Lanka

## Abstract

Sri Lanka is rich in a wide diversity of fruits, but many are underutilized by the people in Sri Lanka despite their nutritional value. This is mainly due to little awareness of the palatability of many fruits and hence low popularity in the market. The present study aimed at providing comparative data on the main biochemical and nutritional parameters of thirty-seven (37) species of fruits grown in Sri Lanka, including 22 underutilized fruits and 15 commonly consumed fruits. The main parameters of the comparison were the contents of ascorbic acid (AA), total vitamin C (TVC), total phenolic content (TPC), total flavonoid content (TFC), total iron (Fe), and antioxidant capacities (ACs). The mean AA, TVC, TPC, TFC, and Fe contents in 100 g of fresh edible portions of fruits ranged from 2.0 to 185.0 mg, 8.1 to 529.6 mg, 12.9 to 2701.7 mg gallic acid equivalent, 0.2 to 117.5 mg quercetin equivalents, and 0.1 to 1.1 mg, respectively. The IC_50_ values in a DPPH assay varied between 0.8 to 1856.7 mg/mL and FRAP values in a FRAP assay ranged from 4.2 to 2070 *μ*mol FeSO_4_/g in the studied fruits. Fruits were ranked based on the levels of the abovementioned biochemical properties. Using this ranking, 12 of the top 15 fruits were underutilized. *Phyllanthus emblica* (Indian gooseberry) is at the top of these underutilized fruits, and *Psidium guajava* (guava) is the best among commonly consumed fruits. These results indicate that underutilized fruits in Sri Lanka can be recommended as high quality and low-cost alternatives for securing nutritional requirements. Hence, underutilized fruits can be promoted as healthy additional fruits in Sri Lanka.

## 1. Introduction

Fruits and plant-based products have been used for food and medicinal purposes since the first human civilizations and indeed since the evolutionary origin of humans [1–3]. There is an emerging trend to consume more fruit on a regular basis, in response to the fact that fruits help to promote human health through supplying essential nutrients, improving immunity functions, and reducing the risk of many noncommunicable diseases (NCDs) such as cancers, diabetes mellitus, arthritis, Alzheimer's disease, Parkinson's disease in both developed, and developing countries [1].

Sri Lanka is a tropical country and a biodiversity hotspot with a wide array of fruits including underutilized and commonly consumed species. Though Sri Lanka has a long history of using fruits as food supplements, nutrient supplements, and for managing health, the fruits termed “underutilized fruits” are those that remain generally unrecognized with little market penetration or popularity within Sri Lanka. This is mainly due to less awareness of their nutritional value and their safety, which is linked to these fruits being less widely grown or commercially cultivated which restricts access to them. Underutilized fruits of Sri Lanka are poorly studied and appreciated. The few studies available, published by various local researchers, have highlighted, for example, the antioxidant capacity of selected fruits grown in Sri Lanka [3–13]. In addition, one recent study has been reported on vitamin C contents and in-vitro antioxidant activities of selected Sri Lankan fruits including some underutilized fruits by Silva and Sirasa [14].

The total vitamin C (TVC) is considered as the sum of ascorbic acid (AA) and dehydroascorbic acid (DHA). Dehydroascorbic acid (DHA) is the oxidized form of the ascorbate, and it has been found that DHA can be reduced reversibly into ascorbate in an enzymatic function and can be stored to increase ascorbate stores in the tissues of guinea pigs; a similar process can be anticipated in humans as well [15]. Since fruits are rich with metabolizing enzymes, recycling of DHA can happen within the fruits, behaving similar to guinea pigs. Scientific studies have yet to be directed to that end. Vitamin C deficiency is a globally significant health issue, particularly in developing countries, with severe deficiency resulting in scurvy. Due to the many pharmacological activities associated with fruits researchers in natural product, chemistry have paid much attention to fruits as preventive measures for highly prevalent NCDs [16, 17]. It is well documented that the phytochemicals present in plant parts including fruits exhibit synergetic pharmacological effects to improve the human immune function which is directly correlated with a reduced risk of NCDs [16]. A suggested cause for many NCDs is excessive oxidative stress in the cells resulting from an imbalance between the generation and the quenching of free radicals, namely, reactive oxygen (ROS) and reactive nitrogen (RNS) species in cells [1, 18, 19]. In that sense, the natural antioxidants such as polyphenols itself identified as nutraceuticals, and ascorbic acids present in the fruits may therefore act as nonenzymatic pathways to quench the harmful radicals and consequently to reduce the excess oxidative stress in cells [20]. Iron deficiency is a very common health issue in the low and middle-income countries including Sri Lanka, and there is a known correlation between dietary iron absorption and ascorbic acid content [21, 22]. Therefore, knowing the data on iron content is equally important along with vitamin C and antioxidant properties of fruits.

.

Although Sri Lanka is a habitat for vast diversity of underutilized fruits, those are less popular among people and hence less market value despite their healthcare and nutritional value. Less availability of data on their nutritional value comparing to the commonly consumed fruits must be the main reason associated with this situation. This should be of great concern for making popular the healthier fruits at low cost. Therefore, this study was carried out with the prime objective of comparing important biochemical parameters which are known to be contributed to human health such as ascorbic acid (AA) content, dehydroascorbic acid (DHA) content, total vitamin C content (TVC), total phenolic content (TPC), total flavonoid content (TFC), total iron (Fe) content, and antioxidant capacities (ACs) of underutilized and commonly consumed fruits grown in Sri Lanka. The purpose is to explore the potential of promoting underutilized fruits among the Sri Lankan community as healthy additional and alternative sources of nutrition and to develop them as economical crops and products.

## 2. Materials and Methods

### 2.1. Chemicals

All the chemicals and reagents including methanol, L-ascorbic acid, 2,6-dichlorophenolindophenol (DCPIP), 2,4-dinitrophenylhydrazine (2,4-DNP), Folin-Ciocalteu's reagent, gallic acid, quercetin (≥95%), 2,2-diphenyl-1-picrylhydrazyl (DPPH), 2,4,6-tris(2-pyridyl)-s-triazine (≥98%) (TPTZ), and iodine used in this study were of analytical grade and purchased from Sigma-Aldrich.

### 2.2. Fruit Samples

Mature fruits from 37 locally grown fruit species (Table 1) were freshly harvested from home-gardens and commercial fruit growers in Sri Lanka. Harvesting or collecting of fruits was done by personal judgment based on experience of the farmer or fruit collector. Subjective criteria for assessing fruit maturity based on physical features of fruits were done. Characteristics of fruits such as skin colour, flesh colour, firmness of the skin, flesh, size, and shape of the fruit and sound when tapped were used. Fruits harvested from different mother plants were kept separately. Just after harvesting, samples were transported in ice boxes to the laboratory. The analyses of those fruit species which are generally harvested at edible maturity (e.g., guava) were started within 24 hours of harvesting, and the other fruit species (e.g., banana) were carried out after keeping them for natural ripening at ambient room temperature.

### 2.3. Preparation of Fruit Samples

After sorting, the fruits were first washed with tap water and secondly with distilled water and then wiped with tissues to dryness. The edible mass of three fruit samples harvested from three different mother plants of the same species were pooled together and a known weight from that edible mass was used to prepare an extract in triplicate. In cases where the fruit is generally consumed as a fruit drink (e.g., *C. sinensis*, *S. caseolaris*), a known weight of juice/pulp was extracted and used for analysis.

### 2.4. Extraction of Vitamin C

Extraction of Vitamin C from plant materials was done according to the reported method (AOAC method 967.21, 45.1.14) (2, 9) as explained by Nielsen [23]. Vitamin C was extracted into a freshly prepared solution containing 3% (*w*/*v*) meta-phosphoric acid and 8% (*v*/*v*) glacial acetic acid. In this method, the groundfruit sample was passed through a muslin cloth and filtrate was collected. The process was done three times and finally collected filtrate was made up to 100 mL with the meta-phosphoric acid-acetic acid solution. The extracts were stored at –10°C until its use for analysis of vitamin C. The extracts were protected from light by covering them with aluminum foil, and all appropriate measures were taken to prevent the loss of ascorbic acid during the extraction process and storage.

### 2.5. Preparation of Methanolic Extract of Fruits to Be Used in the Analysis

Methanolic extracts of fruits were prepared according to the method described by Ikram et al. with slight modifications [24]. The definite amount of fruits was weighed from the homogenized or ground sample of the edible mass of the each fruit species and mixed with 10 mL of 80% (*v*/*v*) of methanol and stirred at 1500 rpm using “AREC Heating Magnetic Stirrer (VELP SCIENTIFICA®)” for an hour. Then, the mixture was centrifuged for 10 min at 5000 rpm (PLC-012E Universal Centrifuge), and supernatant was collected. The extraction process was repeated with the same sample of fruits to make replicates, and the volume was made up to 50 mL. The extracts were stored at –10°C until the analysis was done. These extracts were used for the determination of TPC, TFC, 2,2-diphenyl-1-picrylhydrazyl (DPPH) scavenging activity, and ferric reducing antioxidant power (FRAP) value of fruits.

### 2.6. Sample Analysis

#### 2.6.1. Determination of Total Vitamin C Content (TVC)

Although most past researchers have used the DCPIP titrimetric method to determine the vitamin C content, it is successful only for the ascorbic acid form of TVC and it is also limited to the coloured fruit extract. Due to such limitations in the DCPIP titrimetric method, five other different methods were tested to select the most suitable method for the determination of vitamin C content of fruits in this study. Out of them, we used a slightly modified method to that elaborated by Ranganna [25] adopting from Roe and Oesterling [26]. In this method, 0.5 mL of 3% bromine water (*v*/*v*) was added into 8 mL of sample extract (which is further diluted if necessary) in order to oxidize ascorbic acid into dehydroascorbic acid, and 0.25 mL of 10% thiourea solution (*w*/*v*) was added to remove excess bromine. Then, 2 mL of 2,4-dinitrophenylhydrazine (2,4-DNP) solution (2 g of 2,4-DNP and 4 g of thiourea in 100 mL 4.5 M H_2_SO_4_) was added, and all samples, standards, and blank were kept at 37°C for 3 hours in a thermostatic bath. After cooling in an ice bath for 30 minutes, samples were treated with 10 mL chilled 85% sulfuric acid (*v*/*v*) with constant stirring. Absorbance was measured at 520 nm using a spectrophotometer (HITACHI UH5300 Spectrophotometer). TVC content of each sample was determined as mg per 100 g of fresh weight of the fruit, using a standard curve prepared with L-ascorbic acid, the standard (0.005–0.025 mg/mL).

#### 2.6.2. Determination of Ascorbic Acid (AA) Content

Two methods were used for the determination of AA. In the first method, a titrimetric method described by Suntornsuk et al. [27] was used with slight modifications. Here, an aliquot of 10 mL of sample extract was titrated against iodine (0.005 mol L^–1^) solution, containing 25 mL of 2 N H_2_SO_4_, using a 1% starch (*w*/*v*) as the indicator. Iodine solution was previously standardized using 5 mL of L-ascorbic acid solution (1 mg mL^–1^). In the second method, the AOAC's official titrimetric method (AOAC method 967.21, 45.1.14) (2, 9) was used, as explained by Nielsen [23]. Accordingly, a 5 mL aliquot of extract was titrated with DCPIP reagent until a light but distinct rose pink colour appears and persists for more than 5 seconds. Each analysis was performed in triplicate, and ascorbic acid was expressed as mg of L-ascorbic acid equivalents (AAE) per 100 g of fresh weight of the fruit. In both methods, the iodine and DCPIP solutions were standardized daily with a standard L-AA solution (1 mg mL^–1^). The iodine titration method was applied to all fruit extracts, whereas the DCPIP method was not applied to fruit extracts with a colour (*C. carandas*, *F. inermis*, and *P. granatum*).

#### 2.6.3. Determination of Dehydroascorbic Acid (DHA) Content

DHA content was calculated by subtracting mean AA content by TVC content, and percentage DHA content was determined compared to TVC content.

#### 2.6.4. Determination of Total Phenolic Content (TPC)

The TPC contents of the extracts were determined using the Folin-Ciocalteu's (FC) reagent [28]. Properly diluted fruit extracts (0.5 mL, in triplicate) were stand for 5 min after adding 2.5 mL of FC reagent (10%), followed by addition of 2 mL of Na_2_CO_3_ (7.5% *w*/*v*). The samples were kept in the dark for 30 min, and absorbance was measured at 765 nm. Total phenol content was estimated from a standard curve of gallic acid (0.02–0.1 mg/mL), and TPC contents of fruits were expressed in gallic acid equivalents (GAE) (mg per 100 g of fresh fruit).

#### 2.6.5. Determination of Total Flavonoid Content (TFC)

Aluminum chloride colorimetric method was used to determine the TFC of fruit extracts [29]. Briefly, 1 mL of the fruit extract was mixed with 3 mL of methanol, 0.2 mL of 10% AlCl_3_ (*w*/*v*), 0.2 mL of 1 M potassium acetate, and 5.6 mL of distilled water and stand in the dark for 30 min. The absorbance was measured at 420 nm. TFC of each fruit extract was determined using a standard curve prepared for quercetin (0.01–0.1 mg/mL) and expressed as mg quercetin equivalents (QE) per 100 g of fresh fruit.

#### 2.6.6. DPPH^·^ (2,2′-Diphenyl-1-Picrylhydrazyl Radical) Radical Scavenging Assay

The free radical scavenging activity of fruit extracts was determined by the modified DPPH method [30]. The DPPH solution in methanol (0.06 mM, 3.9 mL) was mixed with 100 *μ*L of fruit extract at different concentrations. The samples were kept in the dark for 30 min, and absorbance was measured at 517 nm. The free radical scavenging activity was expressed as IC_50_ value, calculated using % disappearance vs. concentration plot (concentration means the mg of fruit extract into 1 mL of solution). The % disappearance was calculated from [(*A*_control_–*A*_sample_)/*A*_control_] × 100. *A*_control_ is the absorbance of DPPH without extract, and *A*_sample_ is the absorbance of the DPPH solution containing plant extract.

#### 2.6.7. Ferric Reducing Antioxidant Power (FRAP) Assay

The ferric reducing power of fruit extracts was determined using a modified version of the FRAP assay as originally reported by Benzie and Strain [31]. Three milliliters of freshly prepared FRAP reagent (300 mM acetate buffer (pH 3.6): 10 mM TPTZ (in 40 mM HCl): 20 mM FeCl_3_.6H_2_O in 10 : 1 : 1 ratio) was mixed with 100 *μ*L of diluted sample and absorbance at 593 nm was recorded after 30 min incubation at 37°C. An aqueous solution of FeSO_4_.7H_2_O (100–1200 mM) was used for calibration.

#### 2.6.8. Determination of Total Iron Content (Fe)

*(1) Sample Preparation*. Total iron content was determined for dry digested samples of fruits. Accurately weighed 10–20 g of the edible part of the fruit was first dried in an oven at 100°C and then kept at 450°C in a muffle furnace (Yamato FM-36) for 8 hours or overnight until get ash with white/gray colour. The residue in the crucible was treated with 5 mL of 6 M HCl, and then evaporated on a hot plate. The remaining content was dissolved in 15 mL of 0.1 M HNO_3_ and covered with a watch glass and let stand for 1–2 h. The solution was stirred with a glass rod and then the content was transferred to a volumetric flask and volume was made up to 25 mL with 0.1 M HNO_3_ [32]. These extracts were stored at 4°C and used for the total iron determination.

*(2) Total Iron Determination*. The total iron contents of fruit samples were determined by converting iron to the ferric form using an oxidizing agent, potassium persulphate, and treating with potassium thiocyanate to form red coloured ferric thiocyanate, as elaborated by Ranganna [25]. Five milliliters from the fruit extraction was mixed with 0.5 mL of Conc. H_2_SO_4_, 1.0 mL of K_2_S_2_O_8_ (saturated), and 2 mL of 3N KSCN, and then the volume was made up to 15 mL with deionized water. The absorbance of this solution was measured at 480 nm soon after mixing. Iron standards, ranging from 5 to 25 mg/L, were used for the calibration curve.

### 2.7. Statistical Analysis

One-way analysis of variance (ANOVA) and Tukey's post-hoc tests were used to evaluate the significant differences (*p* < 0.05) of the means between different fruits. The dependent variables considered are TVC, mean AA, TPC, TFC, antiradical power (ARP), FRAP value, and Fe content, and the independent variable was fruit type. Principal component analysis (PCA) was carried out using the IBM SPSS 25.0 statistical software package for Windows (SPSS Inc., Chicago, USA). PCA was performed to classify and discriminate between fruits. In PCA, DPPH radical scavenging activity data were fed as antiradical power (ARP). ARP is reciprocal of IC_50_ (ARP = 1/IC_50_).

## 3. Results and Discussion

### 3.1. Total Vitamin C, Ascorbic Acid, and Dehydroascorbic Acid Contents

#### 3.1.1. Total Vitamin C (TVC) Content

Vitamin C can be defined as the generic term for all compounds exhibiting equivalent biological activity of L-ascorbic acid (AA) and Dehydroascorbic acid (DHA). TVC contents for the studied fruits are given in Table 2. According to that, TVC contents of the fruits tested varied from 8.1 to 529.6 mg/100 g fresh weight (FW). Among the 37 species of fruits studied, the highest TVC content was observed in *P. emblica* followed by *A. marmelos* and apple of *A. occidentale*. The lowest TVC was reported in *S. jambos*. Among the commonly consumed fruits, *P. guajava* (76.2 mg/100 g) had the highest TVC content followed by *C. papaya* (73.2 mg/100 g) and *C. sinensis* (53.6 mg/100 g). Among the underutilized fruits, the TVC content in *P. emblica* was more than 10 times higher than that in *P. guajava* (white). Of the common fruits studied, *G. mangostana* had the lowest TVC content (10.3 mg/100 g). Though total vitamin C content is highly significant when discussing vitamin C content, many previous researchers have not reported TVC, focusing instead on AA in most of studies [14, 33–35].

#### 3.1.2. Ascorbic Acid (AA) Content

In the current study, two methods were applied for the determination of AA to get more accurate results as individual methods have their own limitations such as masking of colour change at the endpoint in fruit extracts with intense fruit colours and interferences of some naturally available substances during the titration, etc. It is difficult to choose a unique method to determine the vitamin C content in all fruits [36]. Although many of past researchers have used only the DCPIP titrimetric method to express the vitamin C content [14, 33, 35], it has some limitations like only the AA form of TVC is measured and difficulties to perform with coloured fruit extracts [36]. Ascorbic acid contents of fruits in this study are given in Table 2, using both I_2_ titration method and the DCPIP titration method. The values varied between 2.2-189.2 and 1.7-181.3 mg AAE/100 g FW in I_2_ method and DCPIP method, respectively. The highest AA content was given for *A. occidentale*, and it is the only fruit that had more than 100 mg of AA per 100 g out of all studied fruits. The most commonly consumed banana had the lowest AA (nearly 2.0 mg/100 g) content. Most of the fruits had lower AA contents, i.e., less than 50 mg/100 g, and only 4 species had AA contents higher than 50 mg/100 g. Out of commonly consumed fruits, *P. guajava* (white) and *C. papaya* had the highest and second-highest AA contents. Although citrus fruits are well known as a rich source of vitamin C, *P. emblica*, *A. occidentale*, *C. papaya*, and *P. guajava* (white) showed significantly higher AA and TVC contents compared to *C. sinensis*.

#### 3.1.3. Dehydroascorbic Acid (DHA) Content

Ascorbic acid (AA) is the main biologically active vitamin C among different forms of vitamin C, and it is reversibly oxidized into DHA, which also involves in biological activities responsible to maintain human health. DHA content of the fruits studied is given in Table 2, and the values are less than 35 mg/100 g except for *A. marmelos* and *P. emblica* which showed 484.8 and 436.7 mg/100 g DHA contents, respectively. The lowest DHA content was observed in *C. carandas* (0.9 mg/100 g). Hernandez, Lobo and Gonzalez [37] has reported lower DHA amounts, than the values obtained in this study for the fruits of *C. sinensis* (2.32 mg/100 g), *M. acuminata* (0.61 mg/100 g), and *A. comosus* (0.36 mg/100 g), but similar values received for *C. papaya* (5.32 mg/100 g) and *M. indica* (5.73 mg/100 g). Gil, Aguayo and Kader [38] also reported similar DHA content for *M. indica* (about 6 mg/100 g) but contrasting results for *A. comosus* and *C. lantanus*. As noted previously, DHA can be converted into reduced form, ascorbate under enzymatic reactions, therefore, presence of higher amount DHA is important to serve as reservoir of ascorbic acid through reversible transformation for the continuous supply and DHA itself plays role in biochemical functions [15]. However, many of previous researchers have not considered both AA and DHA contents when reporting vitamin C contents [34, 37, 38], and our study get highlighted for first reporting of it for Sri Lankan fruits.

### 3.2. Total Phenolic Content, Total Flavonoid Content, and Antioxidant Capacities (ACs)

#### 3.2.1. Total Phenolic Content (TPC)

TPC of studied fruits (Table 3) varied in a broad range as 12.9-2701.7 mg GAE/100 g of FW, and *P. emblica* and *C. lantanus* show the highest and the lowest TPC, respectively. Of common fruits, *P. guajava* (white) (180.6 mg/100 g) showed the highest TPC. Only *P. emblica*, *A. marmelos*, and *D. ovoideum* gave TPC higher than 500 mg/100 g. All the fruits that have very high TPC are underutilized fruits, and the common fruits only have moderate to low TPCs. Polyphenolic compounds in plant including fruits are best known to act as powerful antioxidants and responsible with many pharmacological activities exerted in plants/fruits such as anti-inflammatory and antiglycemic properties [1].

TPCs of fruits grown in Sri Lanka reported by Silva and Sirasa [14] showed similar findings to this study except for gooseberry, star fruit, and pomegranate. However, they have pointed out that the TPC values reported can be higher than the actual values, as they have not used any correction factor for the interfering substances in TPC determination. The TPC values reported by Ellong et al. [39] are comparable with the results in this study except that they have obtained considerably higher TPCs for cashew apple, star fruit, lime, and guava compared.

#### 3.2.2. Total Flavonoid Content (TFC)

The values of TFC are given in Table 3, and they varied between 0.2 to 117.5 mg QE/100 g of FW. The fruits *C. lantanus* and *A. occidentale* (red) showed the lowest and the highest TFCs, respectively. The second highest TFC showed *P. campechiana*, followed by *P. guajava* (white), *P. emblica*, *P. granatum*, and *M. indica*. The TFC values higher than 100 mg/100 g could be observed only in *P. campechiana* and *A. occidentale* (red) which are underutilized fruits, while most of the common fruits had low TFCs (<50 mg/100 g). Flavonoids are very diverse compounds with vast structural diversity as well as great diversity in pharmacological activities such as antioxidant effect and inhibition of cell proliferation [40].

#### 3.2.3. DPPH (2,2′-Diphenyl-1-Picrylhydrazyl Radical) Radical Scavenging Assay

DPPH assay is a widely used *in-vitro* antioxidant assay and based on the ability of DPPH, a stable free radical, to change its colour in the presence of antioxidants. This is a direct and reliable method for determining radical scavenging action of plant extracts. Original DPPH solution is purple colour, and it changed to yellow when plant secondary metabolites reduced it by donating electrons as hydrogen radical. As given in Table 4, IC_50_ values of DPPH assay varied greatly in between 0.8 to 1856.7 mg/mL. According the results, the highest radical scavenging activity (as characterized by the lowest IC_50_) was observed in *E. serratus* followed by *P. emblica*, *A. occidentale* (red), *D. ovoideum*, *A. occidentale* (yellow), *C. cauliflora*, and *P. guajava* (white), and interestingly except *P. guajava* (white) the others are underutilized fruits. Among the common fruits, only *P. guajava* (white) and *M. indica*, and *L. acidissima* and *M. paradisiaca* (embul) showed high radical scavenging activities. The lowest radical scavenging activity (as characterized by the highest IC_50_) was observed in *C. lantanus* followed by *P. americana* and *C. aurantifolia*, and those are commonly consumed fruits. Therefore, these results evidence the greater free radical scavenging activity of locally grown underutilized fruits and comparatively lower activity for commonly consumed fruits.

#### 3.2.4. Ferric Reducing Antioxidant Power (FRAP) Assay

The FRAP assay treats the antioxidants contained in the samples as reductants in a redox-linked colorimetric reaction, and the value reflects the reducing power of the antioxidants, in which antioxidant reacts with Fe^3+^-TPTZ to produce a coloured Fe^2+^ TPTZ complex, which is measured at 593 nm [41]. FRAP values of this study are given in Table 5, and the values range from 4.2 to 2070 *μ*mol FeSO_4_/g FW. *P. emblica* and *P. americana* showed the highest FRAP values, indicating the highest AC. Second highest FRAP value was obtained for *A. occidentale* (red) followed by *A. marmelos* and *A. occidentale* (yellow). Except *P. americana*, all the other fruits are locally grown underutilized fruits.

The only detailed study on antioxidant properties of fruits grown in Sri Lanka has been reported by Silva and Sirasa [14]. The FRAP values reported by Silva and Sirasa [14] are far below the values reported in this study for the most fruits. One possible reason for this deviation could be the loss of phytochemicals at elevated temperatures of 60°C as the authors have used the preparation of fruit extracts at 60°C. Pantelidis et al. [42] have shown that, although cornelian cherry contains high amounts of AA, anthocyanin, and phenolic compounds, their AC measured by the FRAP assay was low. The authors claimed that the reason for this drop is loss of significant part of the AA and anthocyanin during air drying of the sample at 55°C for FRAP assay. As evidence, Piga et al. [43] reported 55% loss in AA and 90% loss in anthocyanins in plum fruits, during the drying at 60°C. Miean and Mohamed [44] had observed that increasing the temperature above 60°C decreased the phenolic amount considerably. At high temperatures, certain phenolics may decompose or combine with other plant components. As this study conducted controlling all the limiting factors, the results obtained in this study are compatible with correct situation of the fruits.

### 3.3. Total Iron (Fe) Content

Total Fe contents of common and underutilized fruits studied varied between 0.1 and 1.1 mg/100 g FW (Table 2). The highest Fe was reported from *P. guajava* (white variety), and the second highest Fe content was recorded for three of the fruit species, *P. granatum*, *A. occidentale*, and *D. ovoideum.* As AA has the ability to enhance the nonheme iron absorption, fruit sources rich in both AA and iron may help in alleviating iron deficiency among people [22]. *P. guajava*, *A. occidentale*, and *P. emblica*, which have high amounts of both AA and total iron, would be potential sources in this regard.

### 3.4. Principal Component Analysis (PCA)

Principal component analysis (PCA) is a statistical dimensional reduction method which employs reducing the larger number of original dependent variables, to a smaller number of orthogonal new set of variables called principal components (PCs) [45]. According to the Kaiser's rule, two principal components were extracted having eigenvalues > 1.0 from the original data set. The Kaiser-Meyer-Olkin measure of sampling adequacy is 0.645. Loading values, eigenvalues, and percent cumulative variance obtained for PCs are as in Table 6. The percent cumulative variance of the first two principal components was almost 70% of the total variance, which meets the general requirement of 70–85% for PCA. Loading values higher than 0.7 are marked in boldface type in “Table 6”. The PC1 correlates strongly with the original variables in descending order as FRAP value, TVC, TPC, and mean AA. These 4 variables of fruits, positively loaded heavily on the PC1, as determined based on the guideline provided by Stevens [46] (factor loading >0.72). However, TFC, ARP, and Fe did not meet Steven's guideline.

**Figure 1:**
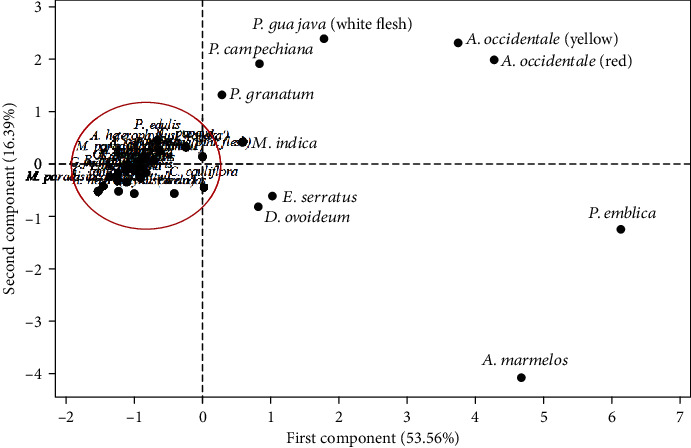
Score plot obtained from principal component analysis.

The score plot resulting from PCA is shown in “Figure 1”, and according to that plot, PC2 has separated all the fruits into two clusters. Most of the fruits are placed on the zero of the PC2 and in the negative side of PC1. The fruits which are in the positive side of PC1 are underutilized fruits except *P. guajava* (White flesh) and *M. indica*. The fruit *P. emblica* has the highest PC1 value, and it can be considered as the best fruit, in terms of studied variables, followed by *A. marmelos*, *A. occidentale* (Red), and *A. occidentale* (Yellow). Interestingly, all these fruits are underutilized fruits. Among commonly consumed fruits, *P. guajava* (White flesh) is found as the best fruit as shown in the positive side of the PC1. The results of the current study emphasizes that the underutilized fruits are with high antioxidant properties comparative to common fruits. These results are quite justifiable with previously reported studies [14, 18, 33, 35].

## 4. Conclusion

This study confirmed that the locally available underutilized fruits *P. emblica*, *A. occidentale*, *A. marmelos*, and *E. serratus* are rich sources of ascorbic acid contents, total vitamin C content, phenolic, flavonoid, iron, and comparably high antioxidant capacity. Among them, *P. emblica* is the highest. Hence, these results suggest that underutilized fruits could be used as a good alternative or addition to common fruits in promoting and securing better health in human populations and reducing the risk of many NCDs and iron deficiency. The production, marketing, and consumption of food depend on many factors other than nutritional quality, such as ease of cultivation and palatability. This work shows the nutritional value of these fruits and suggests the value of efforts in these other areas to encourage greater consumption of these valuable foodstuffs.

## Figures and Tables

**Table 1:** Harvested time, harvested location, and edible part(s) of fruit species used for this study.

Scientific name	Common name(s)	Edible part(s)^1^	Harvested location	Harvested month^4^
Common/utilized fruits				

1*. Ananas comosus* (L.) Murr.	Pineapple (“Murusi”)	WPS	MT^2^, SP^3^	March, 2018
2*. Carica papaya* L.	Papaya (“red lady”)	WPS	MT, SP	March, 2018
3*. Citrullus lantanus* (Thumb.) Matsum & Nakai	Watermelon	WPS	HB, SP	January, 2018
4*. Citrus aurantifolia* (Christm. & Panzer) Swingle	Lime	WPS	HB, SP	March, 2017
5*. Citrus sinensis* (L.) Osbeck	Sweet orange (“Bibila sweet”)	WPS	MN, UP	January, 2018
6*. Garcinia mangostana* L.	Mangosteen	WPS	MT, SP	August, 2018
7*. Limonia acidissima* L.	Wood apple	WP	MT, SP	October, 2017
8*. Mangifera indica* L.	Mango	WPS	MT, SP	May, 2017
9. *Musa paradisiaca* L. AAB “Mysore”	Banana (“Embul”)	WP	MT, SP	November, 2017
10. *Musa paradisiaca* L.AAB, “Silk”	Banana (“Kolikuttu”)	WP	MT, SP	January, 2018
11. *Nephelium lappaceum* L.	Rambutan	WPS	CL, WP	June, 2018
12. *Passiflora edulis* Sims	Passion fruit	WP	HB, SP	November, 2017
13. *Persea americana* Miller.	Avocado	WPS	MT, SP	August, 2018
14. *Psidium guajava* L.	Guava (white flesh)	WF	HB, SP	Jun, 2017
15. *Psidium guajava* L.	Guava (pink flesh)	WF	GL, SP	May, 2018

Underutilized fruits				

16. *Aegle marmelos* (L.) Correa	Bael fruit	WPS	HB, SP	March, 2017
17. *Anacardium occidentale* L.	Cashew apple (red)	WS	MT, SP	April, 2018
18. *Anacardium occidentale* L.	Cashew apple (yellow)	WS	MT, SP	April, 2018
19. *Annona muricata* L.	Sour sop	WPS	HB, SP	August, 2018
20. *Artocarpus heterophyllus* Lam.	Ripened jack fruit (“Wela”, i.e., texture soft or loose when ripe)	WPS	MT, SP	June, 2018
21. *Artocarpus heterophyllus* Lam.	Ripened jack fruit (“Waraka”, i.e., texture somewhat hard when ripe)	WPS	MT, SP	July, 2018
22. *Averrhoa bilimbi* L.	Bilimbi	WF	MT, SP	March, 2017
23. *Averrhoa carambola* L.	Star fruit	WF	MT, SP	February, 2017
24. *Baccaurea motleyana* Mull.-Arg.	Lansone	WPS	GL, SP	July, 2018
25. *Carissa carandas* L.	Jamson	WS	MT, SP	April, 2018
26. *Citrus aurantium* L.	Sour orange	WPS	MN, UP	January, 2018
27. *Cynometra cauliflora* L.	Nam nam	WS	MT, SP	November, 2017
28. *Dialium ovoideum* Thw.	Velvet tamarind	WPS	HB, SP	Oct, 2017
29. *Elaeocarpus serratus* L.	Ceylon olive	WS	MT, SP	March, 2017
30. *Flacourtia inermis* Roxb.	Lovi-lovi, Sapida	WS	MT, SP	July, 2018
31. *Phyllanthus emblica*	Indian gooseberry, Amla	WS	HB, SP	October, 2017
32. *Pouteria campechiana* (Kunth) Baehni	Yellow sapote	WPS	MT, SP	July, 2018
33. *Punica granatum* L.	Pomegranate (local variety)	WPS	HB, SP	September, 2018
34. *Sandoricum koetjape* (Burm. f.) Merr.	Santol/Cotton fruit	WPS	GL, SP	July, 2018
35. *Sonneratia caseolaris* L.	Mangrove apple	WF	MT, SP	June, 2018
36. *Spondias dulcis* Sol. ex Parkinson	Jamaica plum	WPS	HB, SP	February, 2017
37. *Syzygium jambos* (L.) Alston	Rose apple (“Malaysian”)	WS	MT, SP	March, 2017

^1^Edible part(s) of the fruit: WPS: without peel and seed(s); WP: without peel; WS: without seed(s); WF: whole fruit; ^2^Fruits harvested district in Sri Lanka: MT: Matara; HB: Hambantota; CL: Colombo; MN: Monaragala; GL: Galle; ^3^Fruits harvested province in Sri Lanka: SP: Southern province; WP: Western province; UP: Uva province.

**Table 2:** Contents of total Vitamin C (TVC), ascorbic acid (AA), dehydroascorbic acid (DHA), and total iron in common and underutilized fruits of Sri Lanka.

Fruits	TVC (mg/100 g FW)	AA-*I*_2_ (mg AAE/100 g FW)	AA-DCPIP (mg AAE/100 g FW)	DHA (mg/100 g FW)	Classification by TVC contents	Fe (mg/100 g FW)
Common fruits						

*G. mangostana*	10.3 ± 1.1^b^	7.8 ± 0.2^defg^	7.5 ± 0.5^ef^	2.6	Low (<50 mg/100 g FW)	0.2 ± 0.1
*P. edulis*	12.0 ± 0.5^bc^	10.1 ± 0.2^ghi^	10.9 ± 0.1^hi^	1.5		0.3 ± 0.2
*L. acidissima*	12.3 ± 0.2^cd^	9.4 ± 1.0^fgh^	9.0 ± 0.2^fg^	3.1		0.4 ± 0.1
*C. lantanus*	12.4 ± 0.8^cde^	2.8 ± 0.5^a^	3.1 ± 0.4^b^	9.5		0.2 ± 0.1
*P. americana*	14.3 ± 0.6^def^	5.0 ± 0.8^bc^	4.8 ± 0.8^d^	9.4		0.2 ± 0.1
*M. paradisiaca* AAB, “Silk”	15.1 ± 2.5^fg^	2.3 ± 0.5^a^	1.7 ± 0.2^a^	13.1		0.2 ± 0.1
*M. paradisiaca* AAB, “Mysore”	17.0 ± 0.4^gh^	2.2 ± 0.5^a^	1.8 ± 0.1^a^	15.0		0.4 ± 0.1
*P. guajava* (pink flesh)	23.3 ± 0.7^i^	11.0 ± 0.2^hi^	10.0 ± 0.3^gh^	12.8		0.3 ± 0.1
*A. comosus*	31.2 ± 2.4^k^	15.1 ± 0.3^jkl^	12.2 ± 0.4^i^	17.6		0.3 ± 0.1
*C. aurantifolia*	33.3 ± 0.4^kl^	21.1 ± 0.9^mn^	31.1 ± 1.1^mn^	7.2		0.3 ± 0.2
*M. indica*	36.8 ± 0.4^l^	30.8 ± 0.4^op^	28.8 ± 0.2^m^	7.1		0.2 ± 0.1
*N. lappaceum*	49.4 ± 0.6^mn^	18.5 ± 0.2^klm^	11.3 ± 0.3^hi^	34.5		0.2 ± 0.1

*C. sinensis*	53.6 ± 1.2^n^	48.6 ± 1.4^q^	43.9 ± 0.2^o^	7.4	Medium (50–100 mg/100 g FW)	0.1 ± 0.1
*C. papaya*	73.2 ± 1.6^o^	69.5 ± 1.7^r^	64.9 ± 1.8^p^	6.0		0.3 ± 0.0
*P. guajava* (white flesh)	76.2 ± 0.7^o^	68.8 ± 1.0^r^	70.3 ± 0.3^p^	6.7		1.1 ± 0.1

Underutilized fruits						

*S. jambos*	8.1 ± 0.4^a^	6.1 ± 0.3^cd^	5.0 ± 0.2^d^	2.6	Low (<50 mg/100 g FW)	0.1 ± 0.1
*S. caseolaris*	8.4 ± 0.7^a^	7.1 ± 0.4^de^	7.2 ± 0.3^e^	1.2		0.5 ± 0.1
*A. bilimbi*	11.8 ± 0.2^bc^	8.6 ± 0.3^efgh^	7.7 ± 0.4^ef^	3.7		0.2 ± 0.1
*B. motleyana*	14.5 ± 0.5^efg^	4.3 ± 0.6^b^	3.9 ± 0.4^c^	10.4		0.2 ± 0.1
*F. inermis*	18.3 ± 0.5^h^	13.1 ± 0.4^ij^	NA	5.3		0.1 ± 0.0
*A. heterophyllus* (Wela)	18.7 ± 1.1^h^	4.8 ± 0.3^bc^	4.5 ± 0.3^cd^	14.1		0.3 ± 0.1
*P. granatum*	23.6 ± 0.5^i^	14.5 ± 0.5^jk^	NA	9.1		0.9 ± 0.1
*C. carandas*	25.3 ± 0.9^i^	24.4 ± 0.3^no^	NA	0.9		0.2 ± 0.1
*A. carambola*	25.5 ± 0.5^ij^	18.8 ± 0.3^klm^	16.0 ± 0.7^jk^	8.1		0.2 ± 0.1
*S. koetjape*	25.8 ± 0.3^ij^	7.7 ± 0.2^def^	5.3 ± 0.4^d^	19.3		0.4 ± 0.1
*A. muricata*	30.0 ± 1.2^jk^	19.6 ± 0.8^lmn^	18.2 ± 0.5^kl^	11.2		0.2 ± 0.0
*D. ovoideum*	32.9 ± 1.8^kl^	20.3 ± 2.1^mn^	17.5 ± 2.2^jkl^	14.0		0.9 ± 0.1
*C. aurantium*	34.2 ± 0.3^kl^	20.2 ± 0.3^mn^	19.9 ± 0.2^l^	14.2		0.5 ± 0.1
*A. heterophyllus* (Waraka)	34.4 ± 1.4^kl^	16.3 ± 1.3^jklm^	15.0 ± 0.3^j^	18.8		0.3 ± 0.1
*C. cauliflora*	37.9 ± 1.8^l^	31.3 ± 0.2^op^	29.2 ± 1.1^m^	7.7		0.4 ± 0.1
*P. campechiana*	44.8 ± 2.2^m^	32.1 ± 2.0^p^	29.6 ± 0.5^m^	14.0		0.6 ± 0.2
*E. serratus*	45.3 ± 0.3^m^	38.0 ± 0.4^pq^	37.0 ± 0.3^no^	7.9		0.5 ± 0.1

*S. dulcis*	51.2 ± 0.1^mn^	32.0 ± 0.5^p^	29.6 ± 1.5^m^	20.5	Medium (50–100 mg/100 g FW)	0.2 ± 0.1

*A. occidentale* (yellow)	202.3 ± 2.9^p^	189.2 ± 0.9^t^	180.8 ± 0.2^r^	17.3	High (>100 mg/100 g FW)	0.9 ± 0.3
*A. occidentale* (red)	203.3 ± 7.2^p^	188.3 ± 11.9^t^	181.3 ± 5.6^r^	18.5		0.2 ± 0.1
*A. marmelos*	516.6 ± 0.5^q^	31.1 ± 0.7^op^	29.7 ± 0.3^m^	484.8		0.3 ± 0.1
*P. emblica*	529.6 ± 57.5^q^	96.5 ± 3.8^s^	89.3 ± 2.1^q^	436.7		0.7 ± 0.3

Means with different superscript letters in individual column are significantly (*p* < 0.05) different from each other. Data are expressed as Mean ± Standard deviation (*n* = 3). TVC: total vitamin C content; AA-*I*_2_: L-ascorbic acid content determined by Iodine titration method; AA-DCPIP: L-ascorbic acid content determined by DCPIP titration; DHA: dehydroascorbic acid content; AAE: L-ascorbic acid equivalents; FW: fresh weight; NA: not applicable.

**Table 3:** Total phenolic content (TPC), total flavonoid content (TFC), of common and underutilized fruits in Sri Lanka.

Fruits	TPC (mg GAE/100 g FW)	TFC (mg QE/100 g FW)	Classification by TPC's
Common fruits			

*C. Lantanus*	12.9 ± 0.2^a^	0.2 ± 0.1^a^	Low (<100 mg GAE/100 g FW)
*M. paradisiaca* AAB, “Silk”	24.6 ± 0.5^b^	ND	
*G. mangostana*	26.4 ± 0.5^bc^	15.0 ± 0.3^fg^	
*N. lappaceum*	29.9 ± 0.3^cd^	20.7 ± 0.3^hij^	
*A. comosus*	31.3 ± 0.6^d^	14.5 ± 0.5^fg^	
*C. aurantifolia*	49.9 ± 0.9^efg^	10.6 ± 0.5^de^	
*C. papaya*	57.4 ± 1.1^hi^	17.3 ± 0.4^gh^	
*C. sinensis*	66.8 ± 1.4^jk^	13.2 ± 0.2^ef^	
*P. americana*	81.2 ± 1.1^lm^	20.1 ± 0.5^hij^	
*M. paradisiaca* AAB “Mysore”	92.0 ± 1.9^no^	28.4 ± 0.5^klm^	
*P. edulis*	93.5 ± 1.8^no^	50.1 ± 0.2^pqr^	

*L. acidissima*	103.1 ± 6.3^o^	2.4 ± 0.6^b^	Medium (100–500 mg GAE/100 g FW)
*M. indica*	103.8 ± 15.4^o^	62.2 ± 2.8^rs^	
*P. guajava* (pink flesh)	120.6 ± 1.3^p^	43.8 ± 3.5^opq^	
*P. guajava* (white flesh)	180.6 ± 4.3^s^	92.0 ± 0.3^tu^	

Underutilized fruits			

*S. koetjape*	32.7 ± 2.0^d^	14.7 ± 0.4^fg^	Low (<100 mg GAE/100 g FW)
*S. dulcis*	44.7 ± 2.8^e^	23.8 ± 0.8^ijk^	
*B. motleyana*	46.2 ± 0.2^e^	17.4 ± 0.5^gh^	
*A. carambola*	48.1 ± 1.2^ef^	39.2 ± 1.0^nop^	
*A. bilimbi*	53.9 ± 1.4^fgh^	29.4 ± 0.6^klm^	
*S. jambos*	55.3 ± 1.9^gh^	8.4 ± 0.5^cd^	
*C. aurantium*	58.0 ± 1.3^hi^	7.1 ± 0.2^c^	
*A. occidentale* (yellow)	63.3 ± 0.8^ij^	56.0 ± 1.6^qrs^	
*A. heterophyllus* (Waraka)	72.3 ± 0.6^kl^	36.8 ± 0.4^mno^	
*A. muricata*	86.1 ± 0.9^mn^	28.4 ± 0.5^klm^	

*F. inermis*	117.1 ± 2.1^p^	32.6 ± 0.6^lmn^	Medium (100 – 500 mg GAE/100 g FW)
*P. campechiana*	144.6 ± 4.1^q^	113.3 ± 0.4^u^	
*P. granatum*	150.3 ± 1.4^qr^	64.5 ± 2.2^rs^	
*A. occidentale* (red)	153.8 ± 1.6^r^	117.5 ± 0.6^u^	
*S. caseolaris*	164.6 ± 2.1^rs^	6.4 ± 0.4^c^	
*C. carandas*	207.7 ± 2.6^t^	10.6 ± 0.5^de^	
*E. serratus*	212.3 ± 1.3^t^	7.7 ± 0.8^c^	
*A. heterophyllus* (Wela)	221.0 ± 3.9^t^	11.6 ± 0.7^ef^	
*C. cauliflora*	428.5 ± 1.3^u^	26.1 ± 1.0^jkl^	

*D. ovoideum*	804.3 ± 61.1^v^	18.8 ± 1.3^ghi^	High (>500 mg GAE/100 g FW)
*A. marmelos*	1549.2 ± 16.1^w^	56.6 ± 0.4^qrs^	
*P. emblica*	2701.7 ± 2.9^x^	73.9 ± 0.3^st^	

Means with different superscript letters in individual columns are significantly (*p* < 0.05) different from each other. Data are expressed as Mean ± Standard deviation (*n* = 3). TPC: total phenolic content; TFC: total flavonoid content; FW: fresh weight; GAE: gallic acid equivalents; QE: Quercetin equivalents; FW: fresh weight; ND: not detected.

**Table 4:** DPPH assay of common and underutilized fruits in Sri Lanka.

Fruits	DPPH-IC_50_ (mg/mL)	Classification of AC measured by DPPH
Common fruits		

*C. Lantanus*	1856.7 ± 51.3^a^	Very low (IC_50_ > 500 mg/mL)
*P. americana*	1244.3 ± 11.2^b^	

*C. aurantifolia*	467.0 ± 14.7^c^	Low (IC_50_ 100–500 mg/mL)
*G. mangostana*	465.0 ± 1.2^c^	
*A. comosus*	453.3 ± 15.3^cd^	
*P. guajava* (pink flesh)	385.7 ± 31.1^de^	
*N. lappaceum*	347.7 ± 4.9^e^	
*P. edulis*	230.7 ± 8.6^gh^	
*M. paradisiaca* AAB, “Silk”	196.0 ± 15.1^hi^	
*C. sinensis*	167.0 ± 11.1^i^	
*C. papaya*	120.0 ± 10.0^j^	

*M. paradisiaca* AAB “Mysore”	94.8 ± 3.0^k^	High (IC_50_ 20–100 mg/mL)
*L. acidissima*	40.1 ± 0.5^no^	

*M. indica*	12.9 ± 2.1^r^	Very high (IC_50_ < 20 mg/mL)
*P. guajava* (white flesh)	9.8 ± 0.1^s^	

Underutilized fruits		

*S. koetjape*	526.5 ± 5.8^c^	Very low (IC_50_ >500 mg/mL)

*B. motleyana*	276.3 ± 16.5^f^	Low (IC_50_ 100–500 mg/mL)
*C. aurantium*	236.6 ± 7.7^fg^	
*S. jambos*	120.5 ± 16.6^gh^	
*A. heterophyllus* (Waraka)	130.2 ± 10.0^j^	

*P. granatum*	94.4 ± 0.6^k^	High (IC_50_ 20–100 mg/mL)
*A. bilimbi*	93.1 ± 0.2^k^	
*P. campechiana*	70.7 ± 5.5^l^	
*A. muricata*	67.4 ± 0.3^l^	
*A. heterophyllus* (Wela)	51.8 ± 3.5^m^	
*F. inermis*	43.9 ± 2.0^mn^	
*A. carambola*	43.9 ± 0.1^mn^	
*A. marmelos*	39.1 ± 0.2^nop^	
*S. dulcis*	35.1 ± 0.3^op^	
*S. caseolaris*	33.0 ± 1.7^p^	
*C. carandas*	26.4 ± 0.7^q^	

*C. cauliflora*	8.7 ± 0.3^s^	Very high (IC_50_ < 20 mg/mL)
*A. occidentale* (yellow)	8.4 ± 0.3^s^	
*D. ovoideum*	6.6 ± 0.6^t^	
*A. occidentale* (red)	4.4 ± 0.1^u^	
*P. emblica*	1.0 ± 0.1^v^	
*E. serratus*	0.8 ± 0.1^v^	

Means with different superscript letters in individual column are significantly (*p* < 0.05) different from each other. Data are expressed as Mean ± Standard deviation (*n* = 3). DPPH: 2,2-diphenyl-1-picrylhydrazyl; FW: fresh weight; AC: antioxidant capacity.

**Table 5:** FRAP values of common and underutilized fruits in Sri Lanka.

Fruits	FRAP (*μ*mol FeSO_4_/g FW)	Classification of AC measured by FRAP
Common fruits		

*P. americana*	4.2 ± 0.7^a^	Very low (<50 *μ*mol FeSO_4_/g FW)
*C. aurantifolia*	4.8 ± 0.3^a^	
*A. comosus*	6.2 ± 0.2^b^	
*P. edulis*	6.3 ± 0.6^b^	
*G. mangostana*	7.3 ± 2.5^bc^	
*C. sinensis*	11.1 ± 0.7^d^	
*M. paradisiaca* AAB, “silk”	14.5 ± 0.2^ef^	
*M. paradisiaca* AAB “Mysore”	23.9 ± 0.7^h^	
*N. lappaceum*	24.4 ± 0.5^h^	
*L. acidissima*	47.6 ± 0.5^j^	

*C. Lantanus*	79.2 ± 0.9^klm^	Low (50–100 *μ*mol FeSO_4_/g FW)

*C. papaya*	108.3 ± 7.6^no^	High (100–1000 *μ*mol FeSO_4_/g FW)
*P. guajava* (white flesh)	131.5 ± 0.5^o^	
*P. guajava* (pink flesh)	746.6 ± 3.0^r^	
*M. indica*	950.0 ± 78.1^s^	

Underutilized fruits		

*C. aurantium*	8.0 ± 0.4^c^	Very low (<50 *μ*mol FeSO_4_/g FW)
*A. heterophyllus* (Wela)	11.7 ± 0.3^de^	
*S. koetjape*	11.9 ± 0.3^de^	
*A. bilimbi*	14.2 ± 0.3^e^	
*B. motleyana*	18.3 ± 0.7^fg^	
*A. muricata*	20.8 ± 0.4^gh^	
*S. dulcis*	33.2 ± 0.2^i^	
*P. campechiana*	40.3 ± 1.0^ij^	
*A. carambola*	40.4 ± 0.4^ij^	

*C. cauliflora*	63.2 ± 2.9^k^	Low (50–100 *μ*mol FeSO_4_/g FW)
*S. caseolaris*	66.2 ± 2.6^kl^	
*P. granatum*	81.0 ± 0.9^lm^	
*A. heterophyllus* (Waraka)	88.3 ± 1.6^mn^	
*F. inermis*	90.8 ± 0.6^mn^	
*E. serratus*	92.8 ± 2.5^mn^	

*D. ovoideum*	130.0 ± 10.0^o^	High (100–1000 *μ*mol FeSO_4_/g FW)
*S. jambos*	264.2 ± 5.2^p^	
*C. carandas*	527.6 ± 2.5^q^	

*A. occidentale* (yellow)	1388.0 ± 12.5^t^	Very high (>1000 *μ*mol FeSO_4_/g FW)
*A. marmelos*	1634.5 ± 18.6^tu^	
*A. occidentale* (red)	1770.7 ± 26.1^u^	
*P. emblica*	2070.0 ± 61.4^u^	

Means with different superscript letters in individual column are significantly (*p* < 0.05) different from each other. Data are expressed as Mean ± Standard deviation (*n* = 3). FRAP: Ferrous reducing antioxidant power; FW: fresh weight; AC: antioxidant capacity.

**Table 6:** Loading values, eigenvalues, and percent cumulative variance obtained for the two principal components.

Variable	PC1	PC2
TVC	**0.897**	–0.006
Mean AA	**0.722**	–0.260
TPC	**0.793**	–0.503
TFC	0.656	0.473
ARP	0.593	0.578
FRAP	**0.900**	–0.395
Fe	0.451	0.337
Eigenvalue	3.749	1.147
% cumulative	53.558	69.948

TVC: total vitamin C; AA: ascorbic acid; TPC: total phenolic content; TFC: total flavonoid content; ARP: antiradical power; FRAP: ferric reducing antioxidant power.

## Data Availability

The data used to support the findings of this study are available from the corresponding author upon request.
